# Struma ovarii: un cas rare de kyste ovarien géant

**DOI:** 10.11604/pamj.2017.26.223.10621

**Published:** 2017-04-25

**Authors:** Hanane Raiteb, Hicham El Fazazi, Jaouad Kouach, Driss Moussaoui, Mohamed Dehayni

**Affiliations:** 1 Service de Gynéco-Obstétrique, Hôpital Militaire d’Instruction Mohammed V Rabat, Maroc; 2 Faculté de Médecine et de Pharmacie, Université Mohammed V Souissi, Rabat Maroc

**Keywords:** Struma ovarii, thyroïde, goitre ovarien malin, Struma ovarii, thyroid, malignant ovarian goiter

## Abstract

Le struma ovarii est une tumeur rare qui représente 2,7% des tératomes ovariens et 0,01% des tumeurs ovariennes. Elle survient souvent chez la femme au cours de la cinquième décennie et constitue le plus souvent une surprise de l'examen échographique puis histologique. Sa prise en charge est chirurgicale et son pronostic est excellent. Nous rapportons un cas particulier de struma ovarii, vu sa survenue chez une femme jeune, la taille importante de la tumeur et sa présentation radiologique inhabituelle. Nous confrontons ce cas aux données de la littérature.

## Abstract (en)

Struma ovarii is a rare tumor, which accounts for 2.7% of ovarian teratomas and 0.01% of ovarian tumors. It usually affects women in the fifth decades of life and is most often detected incidentally during ultrasound and then confirmed histologically. The gold standard treatment is surgery and prognosis is excellent. We here report the particular case of a young female patient with giant struma ovarii whose radiological features were classified as unusual presentation. We match patient's data with those from the literature.

## Introduction

Le goitre ovarien (ou struma ovarii) est un tératome uni tissulaire de l'ovaire composé majoritairement (plus de 50% de la tumeur) voire exclusivement (goitre ovarien pur) de tissu thyroïdien [1]. Décrit pour la première fois par Von Kalden en 1895, le stuma ovarii est une tumeur rare qui représente 2,7% des tératomes ovariens [2] et 0,01% des tumeurs ovariennes [1]. Nous rapportons un cas qu'on a jugé intéressant par sa survenue chez une patiente jeune, la taille considérable de la tumeur, sa présentation clinique et radiologique non spécifique rendant le diagnostic difficile, nous confrontons ce cas aux données de la littérature.

## Patient et observation

**Figure 1:**
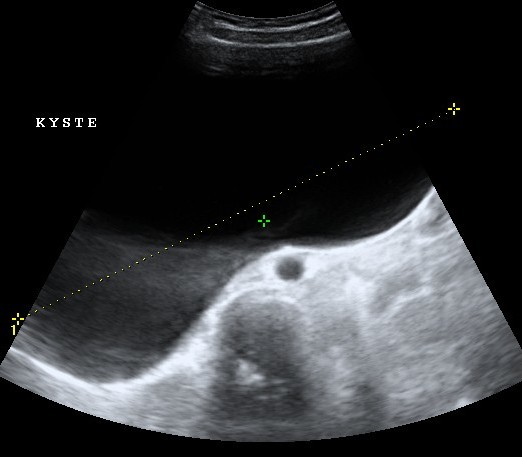
aspect échographique montrant une volumineuse formation kystique pelvienne

**Figure 2:**
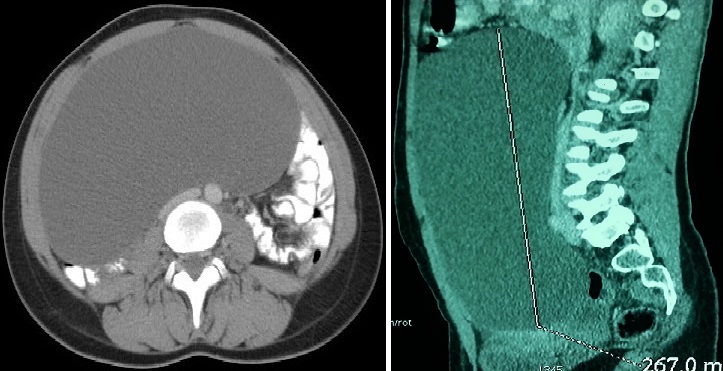
TDM abdomino-pelvienne objectivant une énorme masse kystique bien limitée mesurant 26,7 x 20,4 x 1,5 cm

une augmentation progressive du volume abdominale, des douleurs pelviennes chroniques, compliquée de troubles digestifs faits d'alternance diarrhées, constipation, et de vomissements. L'examen clinique trouvait une énorme masse abdomino-pelvienne qui arrivait à l'hypochondre droit, douloureuse à la palpation, rénitente, elle mesurait 27 cm, l'examen gynécologique est non fait (patiente vierge). L'échographie abdomino-pelvienne mettait en évidence une volumineuse formation kystique médiane et latérale droite probablement d'origine ovarien anéchogène à paroi fine et régulière sans végétation endokystique, elle mesurait 25 x 20 cm refoulant les structures digestives (Figure 1). La TDM abdomino-pelvienne avait objectivé la présence d'une énorme masse kystique bien limitée, de contours nets et réguliers, siège d'une végétation au niveau de sa paroi antéro-inférieure droite (Figure 2). Elle mesurait 26,7 x 20,4 x 1,5 cm sans épanchement péritonéale ni lésion hépatique.

**Figure 3:**
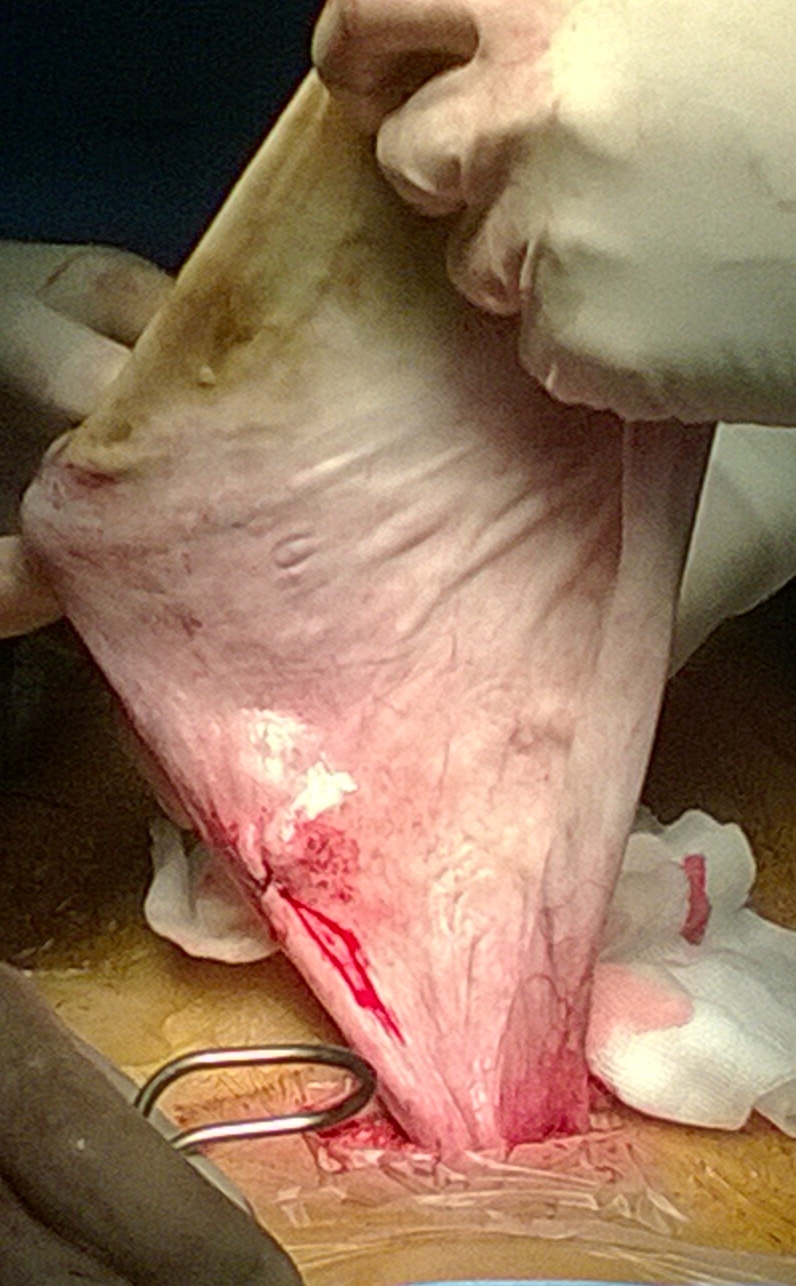
vue opératoire de la paroi kystique

**Figure 4:**
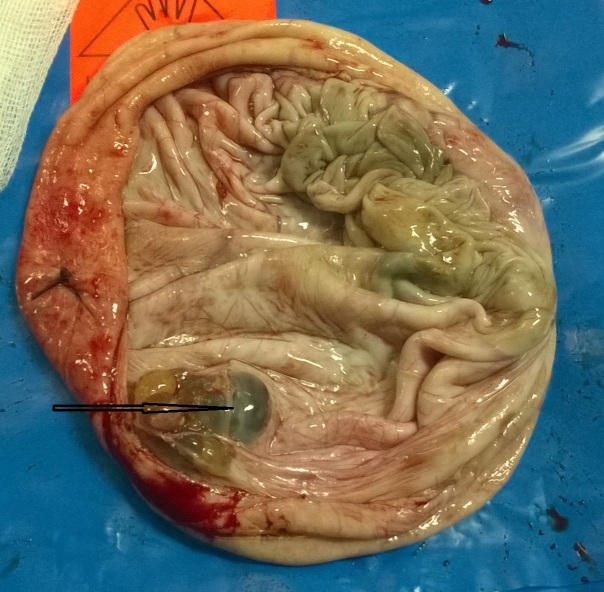
aspect macroscopique de la paroi du kyste avec une végétation endokystique à contenu mucineux

**Figure 5:**
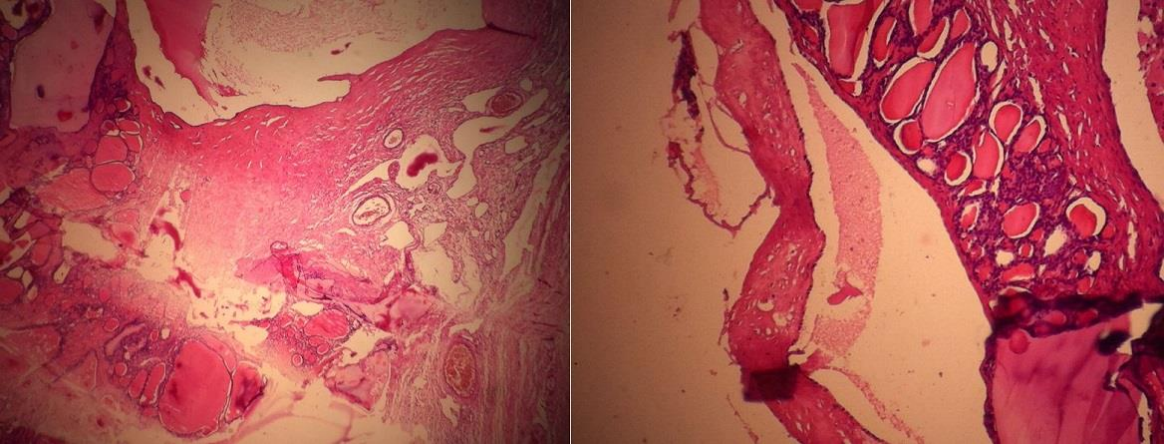
HE Gx10. Aspect microscopique de parenchyme thyroïdien avec des vésicules colloïdes au sein du tissu ovarien

Une mini-laparotomie était réalisée, l'exportation avait objectivé un gros kyste ovarien à paroi lisse qui arrivait au contact du foie sans adhérence, l'annexe gauche et l'utérus étaient sans anomalies. La ponction aspiration du contenu kystique avait ramené 1,5 l de liquide séreux, puis on avait procédé à la kystectomie avec conservation du parenchyme ovarien restant. l'étude macroscopique trouvait une paroi du kyste mesurant 13x13x1 cm (Figure 3 et Figure 4) avec présence d'une végétation endokystique de 2,5x2cm à contenu mucineux, l'étude microscopique montrait une prolifération tumorale composée exclusivement de tissu thyroïdien, d'où le diagnostic de struma ovarii (Figure 5). Les suites opératoires étaient simples.

## Discussion

Le struma ovarii est un tératome mature monodermique rare comportant de façon exclusive ou prédominante du tissu thyroïdien. Il peut subir les remaniements habituels du tissu thyroïdien (adénome, thyroïdite, carcinome) et se compliquer de thyréotoxicose dans 5 % des cas environ [2]. Le pic de fréquence est situé dans la cinquième décennie, l'âge moyen au diagnostic est de 42 ans [3], alors que notre patiente était jeune de 23 ans. Il est souvent unilatéral intéressant l'ovaire gauche dans 63 % des cas, il peut être bilatéral dans 6% des cas [1].

La clinique est le plus souvent frustre, le diagnostic de masse ovarienne étant souvent fortuit ou fait devant l'existence de douleurs pelviennes, d'une masse abdominale ou de troubles du cycle [1]. Une ascite avec parfois un hydrothorax (pseudosyndrome de Meigs) peut être associée à un goitre ovarien [1, 4]. Environ 5 à 8% des goitres ovariens s'accompagneraient d'hyperthyroïdie [1, 3]. En effet, le tissu thyroïdien ectopique peut s'autonomiser [5]. Ce diagnostic doit également être évoqué en cas de scintigraphie thyroïdienne blanche avec un taux de thyroglobuline normal ou augmenté, ou en cas de persistance des symptômes d'hyperthyroïdie après une thyroïdectomie totale [1, 4]. De rares cas découvert lors d'une scintigraphie faite au cours du suivi d'un cancer thyroidien ont été rapporté dans la littérature [6, 7].

En échographie, Le struma ovarii se présente le plus souvent sous forme d'une lésion mixte, kystique et tissulaire, avec des cloisons et végétations [8]. Une hypervascularisation modérée est présente en doppler, en rapport avec une vascularisation du tissu thyroïdien plus riche que celle des composantes tissulaires des autres tératomes [1]. La sémiologie IRM est plus spécifique. Il se présente sous forme d'une lésion hétérogène, mixte, multiloculée, à contours polylobés. Les loci apparaissent de signal variable en rapport avec leur contenu liquide pur (hypersignal T2 et hyposignal T1) ou colloïde (hyposignal T1 et T2) [4, 9] Les cloisons et la portion tissulaire prennent le contraste après injection de gadolinium, en raison de la riche vascularisation du tissu thyroïdien [1, 4]. Il n'existe pas de critère spécifique de malignité en imagerie, en dehors des signes de dissémination métastatique. La particularité de notre observation est la présentation échographique inhabituelle du struma ovarii sous forme kystique pure à paroi fine, la TDM abdominopelvienne, trouvait une masse kystique avec une petite végétation, sans aspect multiloculé ou polylobé. La taille importante de la tumeur fait également l'originalité de notre observation, un seul cas de struma ovarii de 20cm a été rapporté, à notre connaissance, dans la littérature [2]. Le marqueur tumoral CA 125 est classiquement normal dans le contexte de goitre ovarien mais de rares cas d'augmentation ont été rapportés, en cas d'ascitet/ou d'épanchement pleural [4]. Il ne constitue pas un marqueur de malignité du goitre ovarien [4].

L'aspect macroscopique est celui d'une tumeur à composante mixte à contenu muqueux ou gélatineux de couleur marron vert, associée aux autres composants d'un tératome mature dans près de la moitié des cas [1]. L'aspect microscopique retrouve des inclusions de follicules thyroïdiens contenant du colloïde qui sont soit encapsulées, soit irrégulièrement distribuées parmi les autres composants du tératome [1]. L'architecture tissulaire est tout aussi variée que celle de la glande thyroïde. Les critères de malignité du goitre ovarien ont longtemps été controversés, la plupart des auteurs ont retenu comme critères de malignité des goitres ovariens les caractères histopathologiques des carcinomes primitifs de la thyroïde, en dehors de la notion de rupture capsulaire inapplicable à l'ovaire [5]. La transformation maligne de ces tumeurs est extrêmement rare estimée à moins de 1% [10]. Une dissémination métastatique peut survenir dans environ 5% des GOM [1, 10]. Ce taux a été estimé à 23% par Makani et al. [3], d'où la nécessité d'un suivi à long terme. En cas de goitre ovarien bénin, aucun traitement complémentaire à l'ovariectomie unilatérale n'est nécessaire, sauf si une anomalie de la fonction thyroïdienne est associée.

## Conclusion

Le struma ovarii représente une forme rare de tumeur ovarienne, le plus souvent bénigne. Notre observation souligne la difficulté diagnostique devant l'aspect clinique et radiologique aspécifique, le jeune âge de la patiente et la taille inhabituelle de la tumeur. Le traitement est conservateur sous réserve d'un suivi régulier à long terme vu le risque de transformation maligne.
